# The role of on-site pharmacist in residential aged care facilities: findings from the PiRACF study

**DOI:** 10.1186/s40545-023-00587-4

**Published:** 2023-07-03

**Authors:** Ibrahim Haider, Sam Kosari, Mark Naunton, Jane Koerner, Michael Dale, Sundus Nizamani, Rachel Davey

**Affiliations:** 1 Discipline of Pharmacy, Faculty of Health, University of Canberra, Bruce, ACT 2617 Australia; 2 Health Research Institute, Faculty of Health, University of Canberra, Bruce, ACT 2617 Australia

**Keywords:** On-site pharmacists, Residential aged care facilities, Nursing home, Care home, Medication-related problems, Role of pharmacist in aged care, Pharmacist activities

## Abstract

**Background:**

Residents in residential aged care facilities (RACFs) have a high number of medication-related problems. Integrating on-site pharmacists (OSPs) into this setting is a possible solution and is currently gaining traction in Australia and internationally. The Pharmacists in Residential Aged Care Facilities (PiRACF) cluster-randomised controlled trial integrated pharmacists into the RACF care team to improve medication management. The aim of this descriptive observational study is to explore the activities of OSPs when they are integrated into multidisciplinary care team in RACFs.

**Method:**

An online survey tool was developed to record the activities of OSPs in RACFs using the Qualtrics© software. OSPs were asked questions about their activities in RACFs under categories that included description, time spent, outcomes where applicable and who the pharmacists communicated with to undertake the activity.

**Results:**

Six pharmacists were integrated into 7 RACFs. Overall, they recorded 4252 activities over 12 months. OSPs conducted 1022 (24.0%) clinical medication reviews; 48.8% of medication reviews identified and discussed potentially inappropriate medications with prescribers and 1025 other recommendations were made to prescribers. Overall, the prescriber accepted 51.5% of all recommendations made by OSPs. The most frequently accepted outcome was deprescribing of medications (47.5% for potentially inappropriate medications and 55.5% for other recommendations). OSPs performed facility-level activities including staff education (13.4%), clinical audits (5.8%), and quality improvement activities (9.4%). OSPs spent a large proportion of their time communicating (23.4%) extensively with prescribers, RACF’s healthcare team, and residents.

**Conclusion:**

OSPs successfully performed a wide range of clinical activities aimed both at improving residents’ medication regimens, and organisational-level quality improvement. The OSP model presents an opportunity for pharmacists to enhance medication management in the residential aged care setting.

*Trial registration* The trial was registered with the Australian New Zealand Clinical Trials Registry (ANZCTR) (ACTRN: ACTRN12620000430932) on April 1, 2020

## Background

Residents living in residential aged care facilities (RACF) often have multiple co-morbidities and take a high number of medications leading to complex medication management [1, 2]. On average, residents have between 9 and 11 regular medications, increasing the risk of medication-related problems [2–4]. Medication-related problems in the residential aged care setting are widespread, with almost all residents having at least one medication-related problem [5, 6], and up to 68% of residents having at least one regular potentially inappropriate medication (PIM) [7–9]. A meta-analysis of 33 studies has shown PIMs have been linked with an increased risk of hospitalisations [10]. PIMs have also been associated with adverse health outcomes such as the increased risk of falls, cognitive decline, and strokes [11, 12].

Pharmacists usually provide services to RACFs from an off-site pharmacy and only visit to provide clinical services intermittently. This current model may present certain challenges regarding access and communication for pharmacists when it comes to coordinating and following up on medication management recommendations with the multidisciplinary healthcare team in RACFs [1]. Pharmacists may be well placed to improve medication management in RACFs. Pharmacists' expertise in medication management can be utilised to identify and solve residents’ medication-related problems as well as enhance medication quality use and safety at the facility-level. A systematic review investigating factors influencing medication safety indicated that a lack of access to pharmacists as well as inadequate interdisciplinary collaboration impacted negatively upon medication safety in RACFs [13].

In Australia, pharmacists provide services to RACFs through two government-funded services, the residential medication management review (RMMR) and quality use of medicines (QUM) programmes [14]. The RMMR programme is a collaborative medication review service programme allowing an accredited pharmacist to conduct a medication review service for residents in RACFs on a visitational basis following GP (general practitioner) referral. The QUM programme aims to improve medication-related practices at a facility-wide level. Recently, the Australian government conducted a Royal Commission into aged care quality and safety and made recommendations to enhance residents’ care and service quality, including improving medication safety through better access to medication reviews and increasing the role of allied health professionals, including pharmacists [15].

The Pharmacists in Residential Aged Care Facility (PiRACF) study is a cluster-randomised controlled trial to evaluate the effectiveness of a new on-site pharmacist (OSP) model in RACFs. Qualified pharmacists were integrated into RACFs working on-site alongside staff as a member of the RACF care team, with the aim of improving residents’ medication management and reducing medication-related adverse health outcomes. The model was based on a pilot study conducted in 2 RACFs which demonstrated promising findings such as including improvement in medication administration and clinical documentation [16, 17].With the OSP model still in its infancy, the role that pharmacists can play and activities pharmacists can perform in RACFs require exploration. The Australian Commonwealth government announcement to fund $345 million to implement community and on-site pharmacists’ services into RACFs, provides additional rationale to investigate OSP activities in RACFs. Therefore, the objective of this study was to explore the activities of pharmacists when they are integrated as part of the multidisciplinary care team in RACFs.

## Methods

### Study design

This study was an exploratory study conducted as part of the PiRACF study that integrated OSPs into RACFs [18]. An online activity survey was developed to gather information about pharmacists’ daily activities conducted during the PiRACF study to understand how pharmacist perform activities in this role. Each pharmacist was asked to record their activities regularly, optimally daily throughout the PIRACF study.

### The on-site pharmacist model

**Fig. 1:**
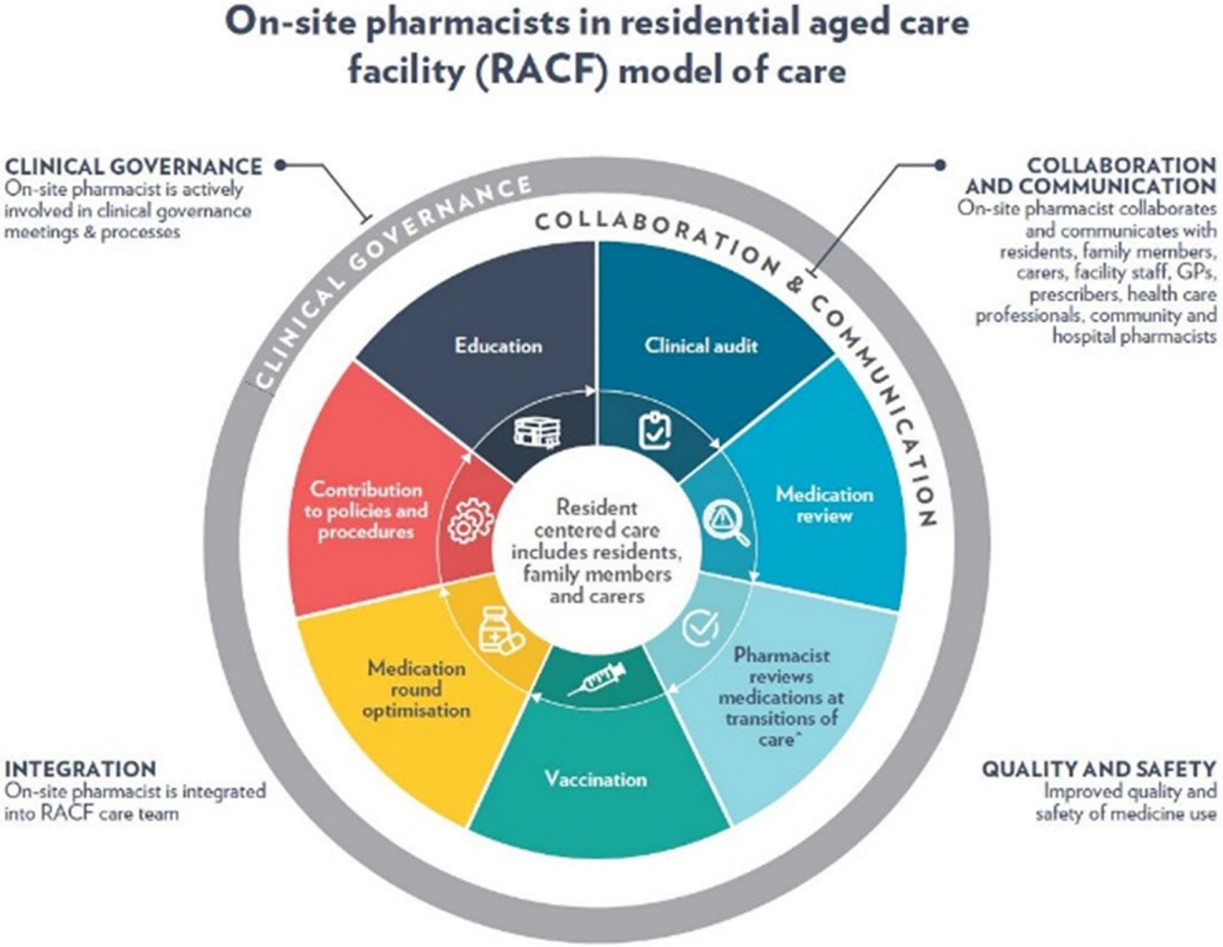
The on-site pharmacist in residential aged care facility model

The OSPs were employed by RACFs to work onsite and be integrated into the multidisciplinary RACF care team for 2–2.5 days a week for a period of 12 months as part of the PiRACF study. The number of hours depended on the size of the facility [18]. OSPs worked within their scope of practice on a prioritised range of activities to improve the quality use of medicines. The intervention was informed by the findings and discussions with RACF managers, GPs, pharmacists, and consumer representatives in the pilot [16–18, 20]. The range of activities involved clinical activities directed at residents, such as clinical medication reviews as well as facility-level activities including clinical audits and contributions to RACF’s policies and procedures (refer to Fig. 1). Pharmacists were given training, resources and documents to assist their work in RACFs [19].

### Data collection

To capture the activities that pharmacists conducted in RACFs, a survey tool was developed to record their activities using the Qualtrics© software. Questions about activities in the survey tool were recorded under categories which collected information about each activity, outcomes (where relevant), time spent and who the pharmacists communicated with to conduct the activity.

Data were initially collected on seven broad categories of pharmacists’ activities in RACFs (please refer to supplementary file 1 for the full survey). The categories of activities in the survey were informed by the pilot study [20]. An additional category was later added for COVID-19-related activities as it became obvious that OSPs would play a role in their respective RACFs response to the COVID-19 pandemic.

### Data analysis

Data were recorded in the Qualtrics© platform and then extracted into Ms Excel© for analysis. Two authors reviewed and then checked the categories of activities and re-categorised if they believed the description of activities fitted another category. Two researchers were consulted in case of recategorising. Any disagreement regarding activity re-categorisation was followed by an independent review with the 3rd researcher until a consensus agreement was obtained. When pharmacists communicated with more than one individual in an activity, the total time of communication was divided by the number of people communicated with to create a per-person time value.

### Research ethics

The study received ethical approval from the University of Canberra Human Research Ethics Committee (ID 2007), ACT Health (2019.LRE.00228) and Calvary Healthcare (2019_ETH13453). The study was conducted in compliance with National Health and Medical Research Council (NHMRC) guidelines. The overarching PiRACF study is registered with the Australian New Zealand Clinical Trials Group (ACTRN: ACTRN12620000430932).

## Results

Seven RACFs participated as intervention sites in the PiRACF trial. Four pharmacists had experience of over 10 years, and the remaining two had 9 years experience or less (one pharmacist worked across two RACFs). Five out of the six participating pharmacists had Medication Management Review accreditation (Table 1).

**Table 1:** Demographic characteristics of pharmacists employed in PiRACF study

Category	Characteristic	*n* of respondents (%)
Gender	Male	1 (16.7)
	Female	5 (83.3)
Age (years)	21 to 30	1 (16.7)
	31 to 40	5 (83.3)
Tertiary qualification	Bachelor’s degree (B. Pharm)	6 (100)
	Postgraduate qualification	2 (33.3)
	MMR accreditation	5 (83.3)
Experience (years)	1–3	1 (16.7)
	4–6	0 (0)
	7–9	1(16.7)
	10 +	4 (66.7)
Accredited immunizer	Yes	4 (66.7)
	No	2 (33.3)

*MMR* Medication Management Review

The pharmacists recorded a total of 4252 activities over the 12 months of the intervention. A breakdown of activities is presented in Table 2. Of the 4252 activities, clinical medication reviews were the most common recorded activity (24%), taking up 1035.8 h, followed by communication (23.4%), administrative tasks related to medication management (19.6%), providing education to RACF staff and residents (13.4%), and quality improvement activities (9.3%).

**Table 2:** Activities of on-site pharmacists in RACFs

Activity	Frequency (%)	Total time in hours (%)	Mean time/activity, minutes ± SD
Comprehensive medication review	1022 (24.0)	1035.8 (27.6)	58.7 ± 43.6
Communication	995 (23.4)	548.2 (14.6)	33.1 ± 30.9
Administrative tasks related to medication management	834 (19.6)	762.9 (20.3)	54.9 ± 59.5
Education	571 (13.4)	469.6 (12.5)	49.3 ± 42.5
Quality improvement	398 (9.4)	400.8 (10.7)	60.4 ± 52.6
Clinical audit	246 (5.8)	357.5 (9.5)	87.2 ± 88.8
Vaccination and related activities	112 (2.6)	125.6 (3.3)	67.3 ± 83.2
COVID-related	63 (1.5)	49.8 (1.3)	47.5 ± 46.3
Other	11 (0.3)	9.3 (0.2)	50.9 ± 61.8
Total	4252 (100)	3759.5 (100)	

Clinical medication reviews involve a systematic review of resident’s medications to identify and resolve medication-related problems. Details of clinical medication reviews conducted by OSPs are presented in Table 3. Pharmacists identified and discussed at least one PIM with the prescriber in 48.8% of all the medication reviews conducted. The most commonly accepted recommendations to PIMs by prescribers were, deprescribing (47.5%), followed by dose reduction (17%). Pharmacists also made recommendations that were not related to PIM medications in their clinical medication reviews, with a total of 1025 recommendations were made, averaging one per medication review. Similar to the recommendations related to PIMs, deprescribing (55.5%) was the most accepted recommendation by the prescribers, but the second most accepted recommendation was to switch to an alternate medication (10.3%). Of all recommendations made by OSPs, the rate of prescriber agreement was 51.5%. While conducting clinical medication reviews, pharmacists worked in collaboration with residents, families, prescribers, and nurses; 43.8% of the communication activities recorded were with GPs, and 29.6% was with the RACF staff.

**Table 3:** Clinical medication review activities

Clinical medication review recommendations and outcomes	Count (% of total)
Number of PIMs identified and discussed with prescribers:	
Number of medication review identifying 1 PIM	310 (30.6)
Number of medication review identifying 2 PIMs	123 (12.1)
Number of medication review identifying 3 PIMs or more	66 (6.5)
Not specified	379 (37.4)
Total	878 (100%)
Recommendations related to PIMs accepted by prescribers	
Medication(s) deprescribed	249 (47.5)
Decrease in dose recommended and accepted	89 (17.0)
Alternative medication(s) recommended and accepted	24 (4.6)
Not specified	162 (30.9)
Total	524 (100%)
Recommendations made not related to PIMs	1025
Recommendations not related to PIMs accepted by prescribers	
Medication(s) deprescribed	253 (55.5)
Alternative medication(s) recommended and accepted	47 (10.3)
Decrease in dose recommended and accepted	81 (17.8)
Increase in dose recommended and accepted	45 (9.9)
Change(s) in dosage form recommended and accepted	30 (6.6)
Total	456 (100%)
Who pharmacists communicated with while conducting medication review	
GP	453 (43.8)
RACF staff	306 (29.6)
Resident	75 (7.2)
Staff at GP reception	54 (5.2)
Residents’ family	49 (4.7)
Community pharmacy	40 (3.9)
Nurse practitioner	40 (3.9)
Hospital	6 (0.6)
Other	12 (1.2)
Total	1035

*PIM* potentially inappropriate medicine

Details of activities of OSPs in RACFs are presented in Table 4. Clinical audits were conducted by pharmacists on multiple topics including psychotropic medications (24.4%), followed by auditing, and updating medication charts (14.6%). Clinical audits were also conducted to identify potentially inappropriate medications, such as the use of PIMs (13.4%), opioids (7.7%), anticoagulants (3.3%), and antimicrobials (2.4%). Pharmacists spent a considerable amount of time (Table 2) on provision of educational activities, totalling 469.6 h (12.5%). Educational activities conducted with RACF staff, included general medication administration (35.9%), opioids/pain management (8.6%), and psychotropics (8.4%).

**Table 4:** Activities of on-site pharmacists in RACFs

Activity	Activity subcategories	Frequency (%)*
Clinical audits	Type of clinical audit:	
	Psychotropics	60 (24.4)
	Medication chart audit	36 (14.6)
	PIMs	33 (13.4)
	Medication management including administration	19 (7.7)
	Opioids	19 (7.7)
	Medications requiring monitoring	14 (5.7)
	Medications prescribed on a PRN basis	9 (3.7)
	Anticoagulants	8 (3.3)
	Residents at high risk of hospitalisation	7 (2.9)
	Antimicrobials	6 (2.4)
	Other	35 (14.2)
Communication	Who pharmacists communicated with:	
	RACF staff	462 (35.7)
	GP (including doctors’ rounds)	206 (15.9)
	Community pharmacy	201 (15.5)
	Resident	131 (10.1)
	Resident’s family	74 (5.7)
	Nurse practitioner	47 (3.6)
	Staff at GP reception	20 (1.5)
	Research staff	18 (1.4)
	Hospital pharmacy	1 (0.1)
	Other	135 (10.4)
	Type of communication used:	
	In person	(54.3)
	Emails	(22.9)
	Phone	(11.7)
	Written communication (i.e. progress notes, communication book)	(3.9)
	Fax	(1.0)
	Text message	(0.20)
	Other	(6.0)
Vaccination	Vaccination and related activities:	
	Staff vaccinated	225 (54.5)
	Residents vaccinated	155 (37.5)
	Other (vaccination-related activities)	33 (8.0)
Education	Education topics:	
	General medication administration (e.g. medication round)	129 (35.9)
	Opioids/pain management	31 (8.6)
	Psychotropics	30 (8.4)
	Specific medical conditions (e.g. dementia/Parkinson’s disease/diabetes)	30 (8.4)
	Inhalers/drops/ointments	18 (5.0)
	Medication crushing	16 (4.5)
	Allergies/side effects/interactions	12 (3.3)
	Medication dosing/timing/expiry/discontinuation	11 (3.1)
	Medication incidents	11 (3.1)
	Cytotoxics	10 (2.8)
	Medication storage	9 (2.5)
	PRNs	9 (2.5)
	Guidelines/policies	8 (2.2)
	Staff training topics (e.g. clinical skills)	8 (2.2)
	Medication changes	7 (1.9)
	Antibiotics	5 (1.4)
	Other (e.g. software/supplements/use of personal protective equipment)	15 (4.2)
Quality improvement	Quality improvement activity:	
	Reviewing RACF policies and procedures and attending relevant meeting	80 (20.2)
	Ward stock related	79 (20.0)
	Medication rounds related	78 (19.7)
	Developing policies and procedures	79 (20.0)
	Controlled medications (Schedule 8 medicines, i.e. opioids)	25 (6.3)
	Reviewing medication incident report	12 (3.0)
	Other	44 (11.1)
COVID-19-related	COVID-19-related activities:	
	Vaccination rollout	17 (27.0)
	Vaccination information (e.g. adverse effects)	12 (19.0)
	Administration of vaccination records (e.g. updating staff COVID vaccination list)	12 (19.0)
	Infection control/outbreak management	9 (14.3)
	COVID administration for facility entry (e.g. risk entry forms)	6 (9.5)
	Staff training/meeting	5 (7.9)
	COVID care (e.g. counselling resident on impacts of lockdown on mental health)	2 (3.2)
Administrative tasks related to medication management	Administrative tasks related to medication management Medication:	
	Clinical administration (e.g. S8 count, recording and destruction, MAC meeting, etc.)	520 (62.4)
	Study-related administration (e.g. meeting with study team, online diary)	301 (36.1)
	Other Admin (e.g. progress notes)	13 (1.6)
Other	Other activities:	
	Other activities (e.g. fire safety training, signing statutory declarations)	11 (100%)

*GP* general practitioner, *PIM* potentially inappropriate medicine, *PRN* pro re nata, *RACF* residential aged care facility

Quality improvement activities are activities pharmacists performed to review or improve medication-related processes within the facility. Among a range of quality improvement activities performed by the pharmacists, the most time, approximately 74.9 h (18.82%), was spent on reviewing and improving existing RACF policies and procedures and attending relevant meetings. Additionally, around 70.1 h (17.60%) were spent on developing new policies and procedures for their RACFs. Another key activity recorded by pharmacists was improving and auditing ward stock with 86.3 h (21.66%) spent overall on this activity. Medication management-related administrative tasks were conducted by pharmacists (19.6% by time, see Table 4). These included counting, recording and destruction of controlled drugs. Other related activities involved attending Medication Advisory Committee and falls meetings and updating progress notes and resident’s records.

OSPs recorded their communications with various members of the multidisciplinary team, as well as with residents and family members. A total of 462 (35.7%) communication activities with RACF staff, 206 (15.9%) activities with GPs (including Drs round) and 201 (15.5%) activities with community pharmacies. Pharmacists also recorded frequent communications with residents 131 (10.1%) and their families 74 (5.7%). Initially, pharmacists conducted influenza vaccinations and related activities, but after the availability of vaccines for COVID-19, the tasks extended to conducting COVID-19 related activities. A total of 125.6 h were spent on vaccinations and related activities by 6 pharmacists (Table 4). The OSPs were actively involved in COVID-19 related activities including assisting in vaccination rollout, recording of vaccinations into the Australian Immunisation Register and provision of information related to COVID-19. A total of 9.3 h were spent in activities that could not be categorised in any of the categories and were classified under Other.

## Discussion

This study describes the activities of OSPs that were included in RACFs as part of the PiRACF cluster RCT conducted over 12 months. OSPs performed a wide range of clinical activities aimed both at the resident and organisational levels. The activities included clinical medication reviews, education, clinical audits, and quality improvement activities. The OSPs spent a large proportion of their time communicating and collaborating with the RACFs healthcare team, residents, and their families. Additionally, pharmacists assisted RACF staff with overall RACF medication management activities including administrative tasks related to medication management, such as attending Medication Advisory Committee and other relevant clinical meetings, auditing and destroying controlled drugs, updating progress notes, and updating resident records. Previous pharmacist-led interventions in RACFs mostly involved the provision of clinical medication reviews as a stand-alone intervention or sometimes followed by a discussion with the GP [21]. In RACFs, residents still experience extensive medication-related problems [9]; integrating pharmacists into aged care facilities help foster collaboration between the healthcare team and as a result may improve medication-related outcomes [20].

Clinical medication review was the central activity performed by OSPs. The medication review involves a systematic assessment of a resident’s medications to optimise therapy and attend to any medication-related problems, with pharmacists following up with residents and prescribers, as necessary. OSPs were successful in identifying and resolving medication-related problems including PIMs. Approximately half of recommendations made by OSPs were accepted by prescribers, with the most frequently accepted recommendation was deprescribing of medications. Other interventions involving medication reviews in RACFs have shown effectiveness in detecting and resolving medication-related problems [2, 21], especially those related to psychoactive drugs [22]. A recent systematic review included eight studies on RMMR interventions, showing effectiveness in identifying medication- related problems; on average, pharmacists identified 2.7–3.9 medication-related problems per RMMR [2]. However, RMMRs are generally underutilised among Australian RACF residents, especially during periods of transitions of care, when medication-related problems often occur [23]. An Australian study of 143,676 residents found only 1 in 5 residents received an RMMR within 90 days [24]. Clinical medication review performed by OSPs may present advantages when compared to other visitational-based medication reviews. The OSP can initiate a medication review when a need arises and without delay, such as during periods of transition when the risk of medication misadventure is elevated. Additionally, OSPs are more likely to develop relationships with residents and staff and to understand their day-to-day issues as they develop, enabling pharmacists to customise medication recommendations based on resident-specific contextual needs and following up on the implementation of recommendations as needed.

OSPs performed a wide range of facility-level activities including provision of education, conducting clinical audits, and implementing quality improvement activities focused on improving medication safety. In addition, pharmacists demonstrated agility in practice by actively assisting in COVID-19 pandemic related activities including vaccination rollouts and providing COVID-19 and vaccination information to staff and residents. To prioritise clinical activities and identify residents needing a clinical medication review, OSPs conducted facility-wide clinical audits of residents to identify those taking PIMs, psychotropics or other high-risk medications. OSPs were also involved in quality improvement activities within facility including reviewing and developing policies and procedure, as well as attending clinical meetings and taking part in the clinical governance of the RACFs. A considerable time was spent by OSPs on providing education to staff on various medication-related topics. It is likely that pharmacist-led educational interventions can improve the knowledge of health care workers in RACFs [21]. In the existing Australian model of practice, visiting contractor pharmacists offer QUM services that are aimed to improve medicines-related practices in RACFs, including participation in medication advisory committees, education, and continuous quality improvement [25]. However, the effectiveness of QUM programme, has not been established [1]. OSPs were successful in performing a wide range of facility-level activities, such as policy development. Integrating an OSP as part of the multidisciplinary team has the potential to improve the quality of medication management practices in RACFs.

Communication plays a crucial role in enhancing decision-making related to medication-related problems and fostering collaboration with healthcare professionals. OSPs documented a significant number of communication activities with various members within the multidisciplinary team as well as residents and their families, with most encounters being in-person. This suggests pharmacists were integrated into the healthcare team and their expertise was being utilised frequently. Collaboration between pharmacists and the RACF's multidisciplinary team can improve current practice models, and pharmacists and residents can be more involved in shared decision-making [26]. On average, OSPs spent longer communicating with GPs than any other member of the healthcare team, showing a high level of engagement with GPs. Working in proximity to the healthcare team within facility, pharmacists can enhance interprofessional communication and collaboration [27]. When performing medication reviews, OSPs communicated frequently with staff at the facility and residents, utilising their presence to collaborate with the multidisciplinary care team to deliver a holistic patient-centred service to residents.

There are limitations to this study. Activities were self-reported by pharmacists and there is a likelihood of reporter bias. Participating pharmacists were experienced and most had the accreditation to conduct RMMRs in Australia. Therefore, the conclusions drawn about OSPs were based on the activities conducted by mostly accredited and experienced pharmacists in the context of a cluster RCT. More research is needed to determine how pharmacists with different levels of experience would perform if integrated into RACFs.

## Conclusion

OSPs in RACFs performed a wide range of clinical activities aimed both at improving residents’ medication regimens, as well as organisational-level quality improvement activities at the RACF level. Pharmacists communicated broadly with the multidisciplinary healthcare team within the facility as well as with residents and their families. In collaboration with the healthcare team, the pharmacist made many recommendations to reduce potentially inappropriate medications. The OSP in aged care model presents an opportunity for pharmacists to be integrated as part of the RACFs healthcare team as medication experts to reduce medication-related problems and help enhance medication management in RACFs.

## Data Availability

The study dataset will not be made publicly available. Only investigators have access to the trial dataset.

## Abbreviations

OSP: On-site pharmacist
RACF: Residential aged care facility
PIM: Potentially inappropriate medication
RMMR: Residential medication management review
GP: General practitioner
QUM: Quality use of medicines
RCT: Randomised controlled trial
NHMRC: National Health and Medical Research Council
